# Molecular prevalence of emerging *Anaplasma* and *Ehrlichia* pathogens in apparently healthy dairy cattle in peri-urban Nairobi, Kenya

**DOI:** 10.1186/s12917-020-02584-0

**Published:** 2020-09-29

**Authors:** Shepelo Getrude Peter, Gabriel Oluga Aboge, Hellen Wambui Kariuki, Esther Gathoni Kanduma, Daniel Waweru Gakuya, Ndichu Maingi, Charles Matiku Mulei, Alfred Omwando Mainga

**Affiliations:** 1 Department of Clinical Studies, Faculty of Veterinary Medicine University of Nairobi, Nairobi, Kenya; 2 Department of Public Health Pharmacology and Toxicology, Faculty of Veterinary Medicine University of Nairobi, Nairobi, Kenya; 3 Department of Microbiology, School of Medicine, University of Nairobi, Nairobi, Kenya; 4 Department of Biochemistry, School of Medicine, University of Nairobi, Nairobi, Kenya; 5 Department of Veterinary Pathology, Microbiology and Parasitology, University of Nairobi, Nairobi, Kenya

**Keywords:** Tick-borne pathogens, *Anaplasma platys*, *Ehrlichia minasensis*, 16S rDNA, Phylogenetics, Molecular diagnostics, Ticks

## Abstract

**Background:**

*Anaplasma* and *Ehrlichia* species are tick-borne pathogens of both veterinary and public health importance. The current status of these pathogens, including emerging species such as *Ehrlichia minasensis* and *Anaplasma platys*, infecting cattle in Kenya, remain unclear, mainly because of limitation in the diagnostic techniques. Therefore, we investigated the *Anaplasma* and *Ehrlichia* species infecting dairy cattle in Nairobi, Kenya using molecular methods.

**Results:**

A total of 306 whole blood samples were collected from apparently healthy dairy cattle. Whole blood DNA was extracted and tested for presence of *Anaplasma* and *Ehrlichia* DNA through amplification and sequencing of the 16S rDNA gene. Sequence identity was confirmed using BLASTn analysis while phylogenetic reconstruction was performed to determine the genetic relationship between the Kenyan isolates and other annotated genotypes available in GenBank. *Anaplasma* and *Ehrlichia* species were detected in 19.9 and 3.3% of all the samples analyzed, respectively. BLASTn analysis of the sequences against non-redundant GenBank nucleotide database revealed infections with *A. platys* (44.8%), *A. marginale* (31%) and *A. bovis* (13.8%). All four sequenced *Ehrlichia* spp*.* were similar to *Ehrlichia minasensis.* Nucleotide polymorphism was observed for *A. platys, A. bovis* and *E. minasensis*. The *Anaplasma* species clustered in four distinct phylogenetic clades including *A. marginale*, *A. platys*, *A. bovis* and some unidentified *Anaplasma* spp. The Kenyan *Ehrlichia minasensis* clustered in the same clade with isolates from America and Australia but distant from *E. ruminantium*.

**Conclusion:**

This study provides the first report of infection of dairy cattle in Kenya with *A. platys* and *E. minasensis*, which are emerging pathogens. We conclude that cattle in peri-urban Nairobi are infected with various species of *Anaplasma* and *E. minasensis*. To understand the extent of these infections in other parts of the country, large-scale screening studies as well as vector identification is necessary to inform strategic control.

## Background

*Anaplasma* and *Ehrlichia* species of the family *Anaplasmataceae* are tick-borne pathogens of livestock with some species known to infect humans and therefore are of both veterinary and public health importance [1]. *Anaplasma* species documented to infect domestic ruminants including cattle are *Anaplasma marginale (A. maginale), A. centrale, A. ovis, A. bovis*, *A. phagocytophilum* and more recently *A. platys* [2, 3]. *Anaplasma marginale* transmitted by several tick vectors including some *Rhipicephalus* (*boophilus*) species is the most common in Kenya causing a severe hemolytic disease in the ruminants. *Anaplasma centrale* whose only known vector is *Rhipicephalus simus* [4] causes mild anaplasmosis in cattle [5] but has not been reported in Kenya possibly because the tick vector is absent. *Anaplasma bovis* transmitted by various species of *Amblyomma* and *Rhipicephalus* ticks [6] causes sub-clinical disease in cattle and has been recently been reported in indigenous calves in Kenya [7].

*Anaplasma platys* transmitted by *Rhipicephalus sanguineus* typically infects dogs resulting in canine infectious cyclic thrombocytopenia [8]. However, *A. platys* has also been isolated from cattle neutrophils [9]. To date, there is no information on cattle infection with this pathogen in Kenya. *Anaplasma phagocytophilum* is a zoonotic species that has been documented to infect cattle resulting in fever, respiratory signs*,* reduced milk production and infertility [10]. *Anaplasma phagocytophilum* was detected recently in questing ticks in a National park in Kenya [11]. For *Ehrlichia* species*, E. ruminantium* transmitted by ticks in the genus *Amblyomma* and the emerging *Ehrlichia minasensis* (*E. minasensis*) are the only species in that genera known to infect cattle [12, 13]. Infections of cattle with *E. ruminantium* and *E. minasensis* are mainly characterized by severe fever, anemia, thrombocytopenia and enlarged lymph nodes [14].

Infections of cattle with the pathogenic species of *Anaplasma* and *Ehrlichia* cause mortalities and morbidities with subsequent losses in production of dairy and beef products. The losses usually result in marked economic impact to dairy and beef farmers in the tropical and sub-tropical regions [15] contributing to poverty in the affected households.

In Kenya including the peri-urban Nairobi, anaplasmosis and theileriosis are some of the tick-borne diseases (TBDs) known to cause economic losses to dairy farmers [16, 17]. Although ehrlichiosis caused by *Ehrlichia ruminantium* has been reported in Kenya [7] and approximately 150 million animals are at risk of infection in Africa [18], quantification of its economic impact in Kenya has not been evaluated possibly because of the difficulty in confirming the diagnosis and the fact that this infection commonly co-infects with other TBDs such as East Coast Fever and anaplasmosis [7]. For a long time, the identification of cattle infected with pathogenic *Anaplasma* and *Ehrlichia* in Kenya has been based mainly on questionnaire data, clinical signs, microscopic examination [16, 19] and serological tests [20, 21]. Nevertheless, few studies in Kenya have used molecular techniques such as reverse line blot, polymerase chain reaction and sequencing for the confirmation and characterization of the tick-borne pathogens infecting cattle [7, 22]. Previously, there had been clinical cases reported to the University of Nairobi Veterinary Hospital, Kenya presenting with unspecific clinical signs such as unthriftness and loss of body conditions. On screening of cattle from the areas where the clinical cases had originated, *E. ruminantium* was identified by antigen detection using Enzyme-linked Immunosorbent Assay (ELISA) [23]. Additionally, microscopic examination of blood from these cattle revealed that some of them had *Ehrlichia*-like inclusion bodies in the white blood cells suggesting infections with other unknown potentially pathogenic haemoparasites [23]. Hence, there was need to identify and characterize those *Ehrlichia*-like inclusion bodies observed in these animals.

Therefore, to further characterize the *Anaplasma* and *Ehrlichia* species infecting dairy cattle in Kenya, this study used molecular tools to determine the prevalence and genetic profiles of various species circulating in apparently healthy dairy cattle in peri-urban Nairobi. Sequence identities and phylogenetic analysis were used to determine the presence of emerging pathogens such as *A. platys* and *E. minasensis*. Subsequently, we provide information that will aid in the understanding of molecular epidemiology of these emerging pathogens of livestock in Kenya and possible epidemiological factors contributing to their spread.

## Results

### Prevalence of *Anaplasma* and *Ehrlichia* species

**Fig. 1:**
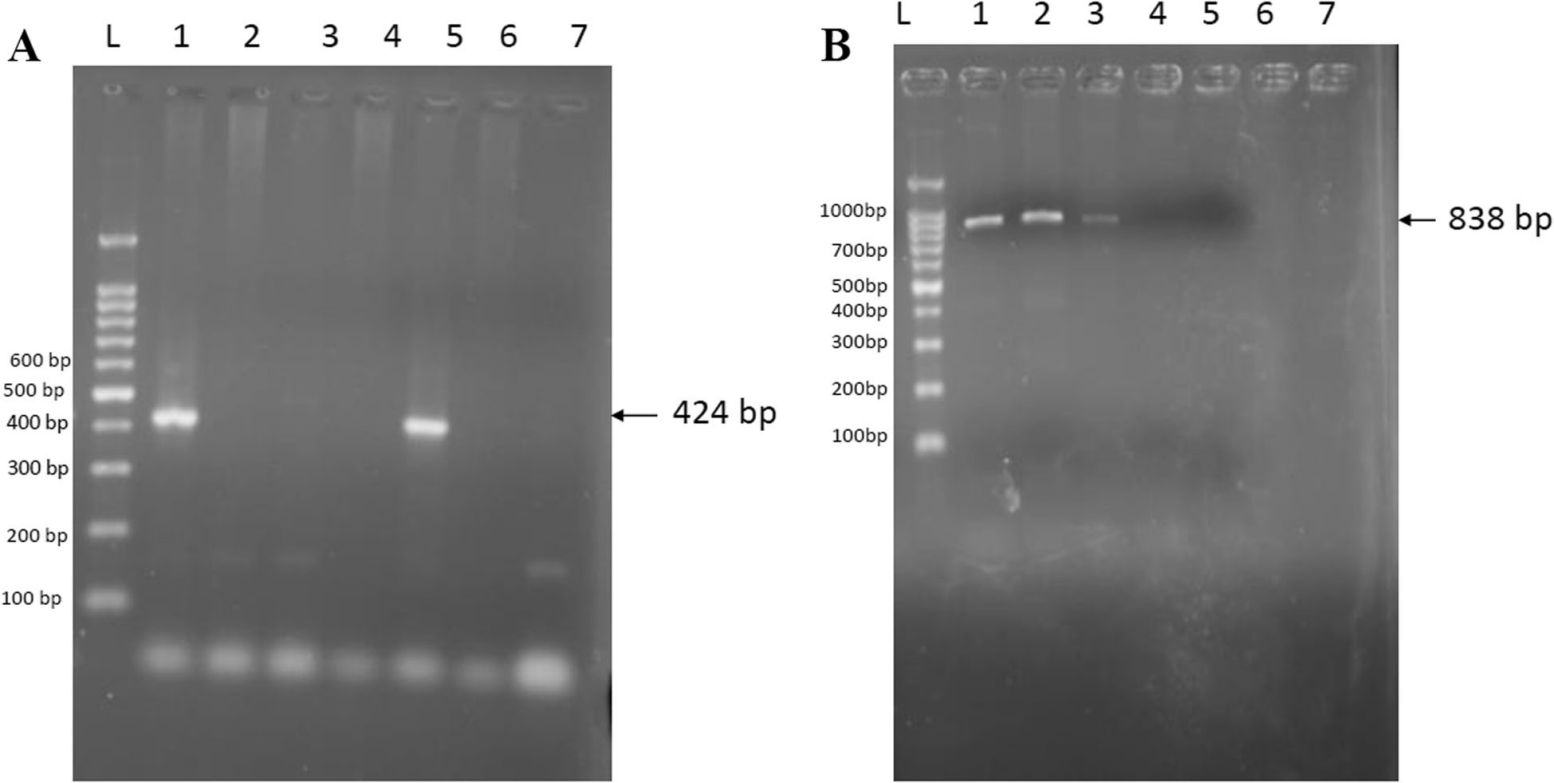
Some of the PCR amplicons of *Anaplasma and Ehrlichia* 16S rDNA gene **a**) PCR product of *Anaplasma* species. Lane L: molecular ladder, lane lanes 1 and 5: positive samples showing amplicon at approximate 424 bp, lanes 2, 3, 4 and 6: no amplicons were observed, 7: negative control. **b**. PCR product of *Ehrlichia* species. Lane L: molecular ladder lane, lanes 1, 2 and 3: positive samples showing amplicon band at approximate 838 bp, lanes 4, 5 and 6: no amplicons were observed, 7: negative control

Of the 306 blood DNA samples analyzed, 61 (19.9%) [95% CI 15.6–24.9] were PCR positive for *Anaplasma* species while 10 (3.3%) [95% CI 1.6–5.9]) were positive for *Ehrlichia*. The *Anaplasma* species yielded a specific band corresponding to 424 bp (Fig. 1a) while primers targeting the 16S rDNA gene of *Ehrlichia* species produced a specific band corresponding to the expected size of 838 bp (Fig. 1b). The distribution of the positive samples in different sub-counties is shown in Table 1. The highest numbers of both *Anaplasma* 34 (55.7) and *Ehrlichia* 7(70) infections were found in Kasarani-Ruai Sub-County while Lang’ata had the least number of cattle positive for *Anaplasma* 6(9.8) infection. *Ehrlichia* infections were however not detected in cattle in Dagorreti Sub-County.

**Table 1:** Distribution of *Anaplasma* and *Ehrlichia* spp. positive cattle in the four sub-counties of Nairobi County

Sub-County	No. of *Anaplasma* spp. (%) [95% CI]	No. of *Ehrlichia* spp*.* (%) [95% CI]
Kasarani-Ruai	34 (55.7) [42.4–68.5]	7 (70.0) [34.8–83.3]
Westlands	11 (18.0) [9.4–29.9]	1 (10.0) [0.2–44.2]
Langata	6 (9.8) [3.6–20.2]	2 (20.0) [2.0–55.6]
Dagorreti	10 (16.4) [8.2–28.0]	0 (0.0) [0]
Total	61 (100)	10 (100)

### Sequence identities of the *Anaplasma* and *Ehrlichia* species detected

Twenty-nine PCR amplicons for *Anaplasma* and four for *Ehrlichia* were sequenced for confirmation of the identities of the detected pathogens. BLASTn analysis revealed that majority, 13(44.8%) of the *Anaplasma* 16S rDNA sequences were similar to *A. platys* with sequence identity of between 98.72 and 100% to annotated sequences in Genbank. Nine (31%) of the sequences were similar to *A. marginale* with a sequence identity of between 99.07 and 100%. Other sequences matched *A. bovis* 4(13.8%) with sequence identity of between 99.28 and 100% and unidentified *Anaplasma* species 3(10.3%) sequence identity of 97.85 to 100% (Table 2). All the four *Ehrlichia* sequences were similar to those of *E. minasensis* revealing a sequence identity of between 99.42 and 100% (Table 3).

**Table 2:** *Anaplasma* species detected by BLASTn analysis of 16S rDNA gene sequences of the Kenyan isolates

Isolate	Our accession number	Matching sequence	Accession no. of highest match	E-value	% Identity
20	MT163376	A. *platys*	MN630836.1	0.0	100.00
46	MT163377	A. *platys*	MK408655.1	0.0	99.28
79	MT163378	*A. platys*	MN630836.1	0.0	100.00
85	MT163379	A. *platys*	MN630835.1	0.0	99.73
97	MT163380	A. *platys*	MN401150.1	0.0	99.76
100	MT163381	A. *platys*	MK408655.1	0.0	99.77
117	MT163382	A. *platys*	MN630836.1	0.0	100.00
173	MT163387	A. *platys*	MN630836.1	6^e-154^	98.72
175	MT163388	A. *platys*	MN401150.1	0.0	99.51
268	MT163383	A. *platys*	MN401150.1	0.0	100.00
318	MT163384	A. *platys*	MN159065.1	0.0	100.00
381	MT163385	A. *platys*	MN630836.1	0.0	100.00
425	MT163386	A. *platys*	MN861060.1	0.0	99.76
127	MT163438	A. *marginale*	MK310488.1	0.0	99.76
139	MT163439	A. *marginale*	MK310488.1	0.0	100.00
159	MT163440	A. *marginale*	MK016525.1	0.0	100.00
168	MT163441	A. *marginale*	MK310488.1	0.0	100.00
171	MT163442	A. *marginale*	MK310488.1	0.0	100.00
172	MT163443	A. *marginale*	MK016525.1	0.0	99.07
239	MT163444	A. *marginale*	MK310488.1	0.0	99.04
243	MT163445	A. *marginale*	MK016525.1	0.0	100.00
342	MT163446	A. *marginale*	MK310488.1	0.0	99.77
39	MT160355	A. *bovis*	MT036513.1	0.0	100.00
75	MT160356	A. *bovis*	MK028574.1	0.0	100.00
86	MT160357	A. *bovis*	MT036513.1	0.0	99.28
326	MT160358	A. *bovis*	MK028573.1	0.0	100.00
103	MT163684	Unidentified *Anaplasma* spp.	KY924885.1	0.0	100.00
112	MT163683	Unidentified *Anaplasma* spp.	KY924884.1	0.0	99.18
166	MT163685	Unidentified *Anaplasma* spp.	KY924884.1	0.0	97.85

**Table 3:** *Ehrlichia* species detected by BLASTn analysis of 16S rDNA gene sequences

Isolate	Our accession number	Matching sequence	Accession no. of highest match	E-value	% Identity
32E	MT163429	*E. minasensis*	MH500005.1	0.0	100.00
86E	MT163430	*E. minasensis*	MH500005.1	0.0	99.42
175E	MT163431	*E. minasensis*	MH500005.1	0.0	99.71
181E	MT163432	*E. minasensis*	MH500005.1	0.0	100.00

### Phylogenetic analysis

**Fig. 2:**
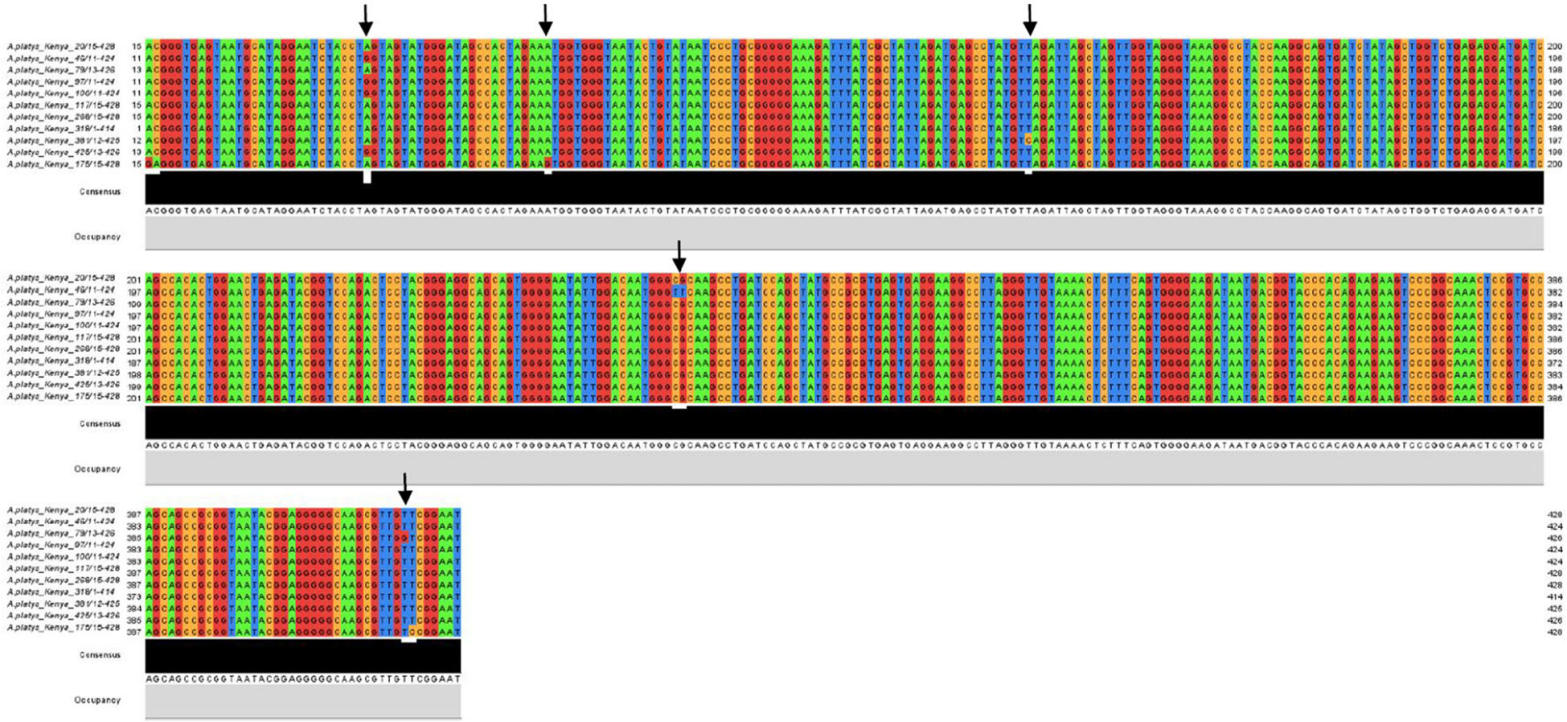
Multiple sequence alignment of *A. platys* 16S rDNA, indicating areas of sequence nucleotide polymorphism (black arrows). Numbers at the ends of each sequence indicate nucleotide lengths while the isolate names are indicated on the far left end of the nucleotide sequences

Multiple sequence alignment was done to assess the genetic similarity of the Kenyan isolates. The nucleotide sequences of three *A.bovis* isolates were conserved while one (MT160357) had three nucleotide polymorphisms at position 267, 268 and 332 (Table 4). *Anaplasma platys* sequences showed divergence of upto 4% (Table 5) with regions of nucleotide polmorphism (Fig. 2). The *A. platys* sequences MT163377 and MT163388 indicated multiple single nucleotide polymorphism while the other five isolates showed a single nucleotide polymorphism (SNP) (Table 4). All the *Anaplasma marginale* sequences from this study were however highly conserved sharing 97.6 to 100% nucleotide similarity (< 2.5% divergence) (Table 6). For *E.minasensis,* two isolates had conserved sequences while isolates MT163430 and MT163431 appeared to be genetically different showing multiple SNPs (Table 4). The multiple sequence nucleotide polymorphisms observed in the Kenyan isolates of *A.bovis*, *A.platys* and *E.minasensis* indicate that various strains of the pathogens may exist in the cattle in peri-urban Nairobi.

**Table 4:** Nucleotide polymorphisms among 16SrDNA sequences of *A. platys*, *A. bovis* and *E. minasensis* Kenyan isolates

		^**a**^Nucleotide position – *Anaplasma platys*							
Isolate	^**b**^Accession no.	1	30	55	118	257	258	407	408
20	MT163376	A	A	A	T	C	G	T	T
46	MT163377 - MSNP	*	G	*	*	T	T	*	*
79	MT163379 -SNP	*	*	*	*	*	*	G	*
97	MT163380 -SNP	*	G	*	*	*	*	*	*
100	MT163381 -SNP	*	G	*	*	*	*	*	*
175	MT163388 - MSNP	G	G	G	*	*	*	*	C
381	MT163385 -SNP	*	*	*	C	*	*	*	*
		^**a**^Nucleotide position – *Ehrlichia minasensis*							
Isolate	^**b**^Accession no.	1	130	257	652				
32E	MT163429	G	C	A	G				
181E	MT163432	*	*	*	*				
86E	MT163430 - MSNP	*	T	C	T				
175E	MT163431-SNP	A	*	*	*				
		^**a**^Nucleotide position – *Anaplasma bovis*							
Isolate	^**b**^Accession no.	267	268	332					
39	MT160355	C	G	G					
75	MT160356	*	*	*					
326	MT160358	*	*	*					
86	MT160357 - MSNP	T	T	A					

Key: ^a^Numbers denotes the nucleotide position on the sequence. Conserved nucleotide positions relative to the first sequence are indicated using asterisks while the specific nucleotide is indicated where a substitution occurred. MSNP- Multiple single sequence polymorphism, SNP-Single nucleotide polymorphism. Nucleotides: T-thymine, C-cytosine, G-guanine, A-adenine. ^b^Genbank Accession numbers

**Table 5:** Pairwise percent identity matches of 16SrDNA sequences of *A. platys* isolated from cattle in Kenya

Isolates	ApN173	ApN46	ApN268	ApN20	ApN117	ApN381	ApN425	ApN100	ApN85	ApN97	ApN175	ApN79	ApN318
**ApN173**	100.0	97.8	96.2	96.2	96.8	98.1	98.4	98.4	98.4	98.4	95.9	98.1	98.4
**ApN46**	97.8	100.0	97.6	97.6	98.1	99.3	99.5	99.5	99.5	98.6	96.7	98.1	98.3
**ApN268**	96.2	97.6	100.0	99.8	99.8	98.4	98.1	98.1	97.9	97.2	98.1	97.2	99.0
**ApN20**	96.2	97.6	99.8	100.0	99.8	98.4	98.1	98.1	97.9	97.2	98.1	97.2	99.0
**ApN117**	96.8	98.1	99.8	99.8	100.0	98.8	98.6	98.6	98.4	97.6	98.6	97.6	99.0
**ApN381**	98.1	99.3	98.4	98.4	98.8	100.0	99.8	99.8	99.7	98.8	97.4	98.8	99.0
**ApN425**	98.4	99.5	98.1	98.1	98.6	99.8	100.0	100.0	100.0	99.1	97.2	98.6	98.8
**ApN100**	98.4	99.5	98.1	98.1	98.6	99.8	100.0	100.0	100.0	99.1	97.2	98.6	98.8
**ApN85**	98.4	99.5	97.9	97.9	98.4	99.7	100.0	100.0	100.0	100.0	97.6	99.7	99.7
**ApN97**	98.4	98.6	97.2	97.2	97.6	98.8	99.1	99.1	100.0	100.0	97.6	99.5	99.8
**ApN175**	95.9	96.7	98.1	98.1	98.6	97.4	97.2	97.2	97.6	97.6	100.0	97.6	99.0
**ApN79**	98.1	98.1	97.2	97.2	97.6	98.8	98.6	98.6	99.7	99.5	97.6	100.0	99.8
**ApN318**	98.4	98.3	99.0	99.0	99.0	99.0	98.8	98.8	99.7	99.8	99.0	99.8	100.0

Key: *Abbreviation*: *ApN Anaplasma platys* Nairobi, followed by the isolate number

**Table 6:** Pairwise percent identity matches of 16SrDNA sequences of *A. marginale* isolated from cattle in Kenya

Isolates	AMN239	AMN172	AMN168	AMN139	AMN159	AMN171	AMN243	AMN127	AMN342
**AMN239**	100.0	99.1	97.6	97.6	97.6	97.6	97.6	98.1	98.3
**AMN172**	99.1	100.0	98.4	97.6	97.6	97.6	97.6	98.8	99.1
**AMN168**	97.6	98.4	100.0	97.6	97.6	97.6	97.6	99.1	99.1
**AMN139**	97.6	97.6	97.6	100.0	100.0	100.0	100.0	98.1	98.3
**AMN159**	97.6	97.6	97.6	100.0	100.0	100.0	100.0	98.1	98.3
**AMN171**	97.6	97.6	97.6	100.0	100.0	100.0	100.0	98.1	98.3
**AMN243**	97.6	97.6	97.6	100.0	100.0	100.0	100.0	98.1	98.3
**AMN127**	98.1	98.8	99.1	98.1	98.1	98.1	98.1	100.0	100.0
**AMN342**	98.3	99.1	99.1	98.3	98.3	98.3	98.3	100.0	100.0

Key: The numbers denote the nucleotide identity rates found between the sequences. *Abbreviation*: *AMN Anaplasma marginale* Nairobi, followed by the isolate number

**Fig. 3:**
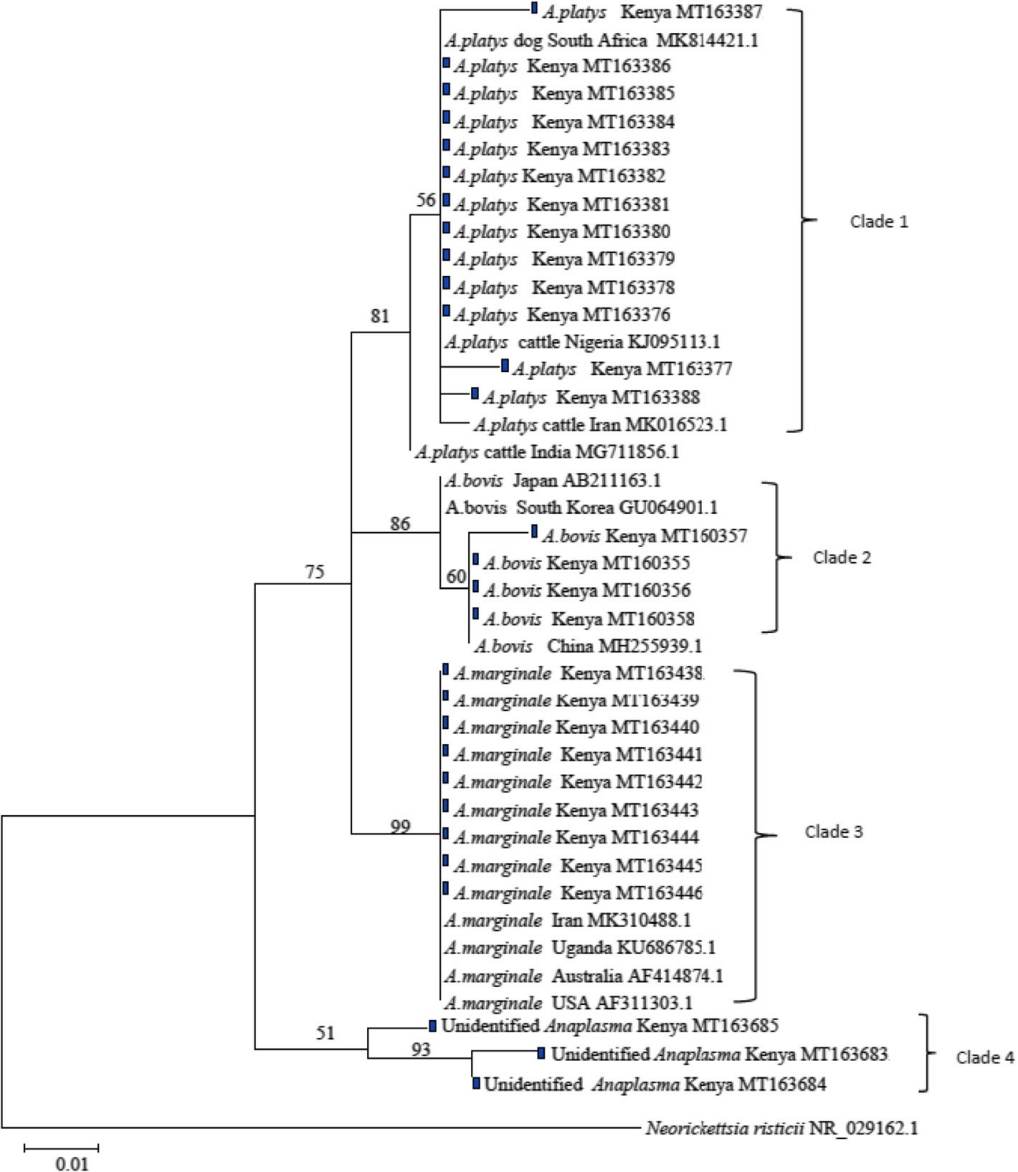
Maximum Likelihood tree of *Anaplasma* spp*.* constructed using partial sequences of 16S rDNA gene. The tree is drawn to scale with branch lengths measured in the number of substitutions per site. The analysis involved 29 nucleotide sequences from this study and 12 others obtained from Genbank. The tree shows the phylogenetic relatedness of *Anaplasma* isolates obtained from cattle blood in Kenya marked with dark box and sequences from other countries. *Neorickettsia risticii* was used as the outgroup. Sequence accession numbers are given at the end of each isolate

**Fig. 4:**
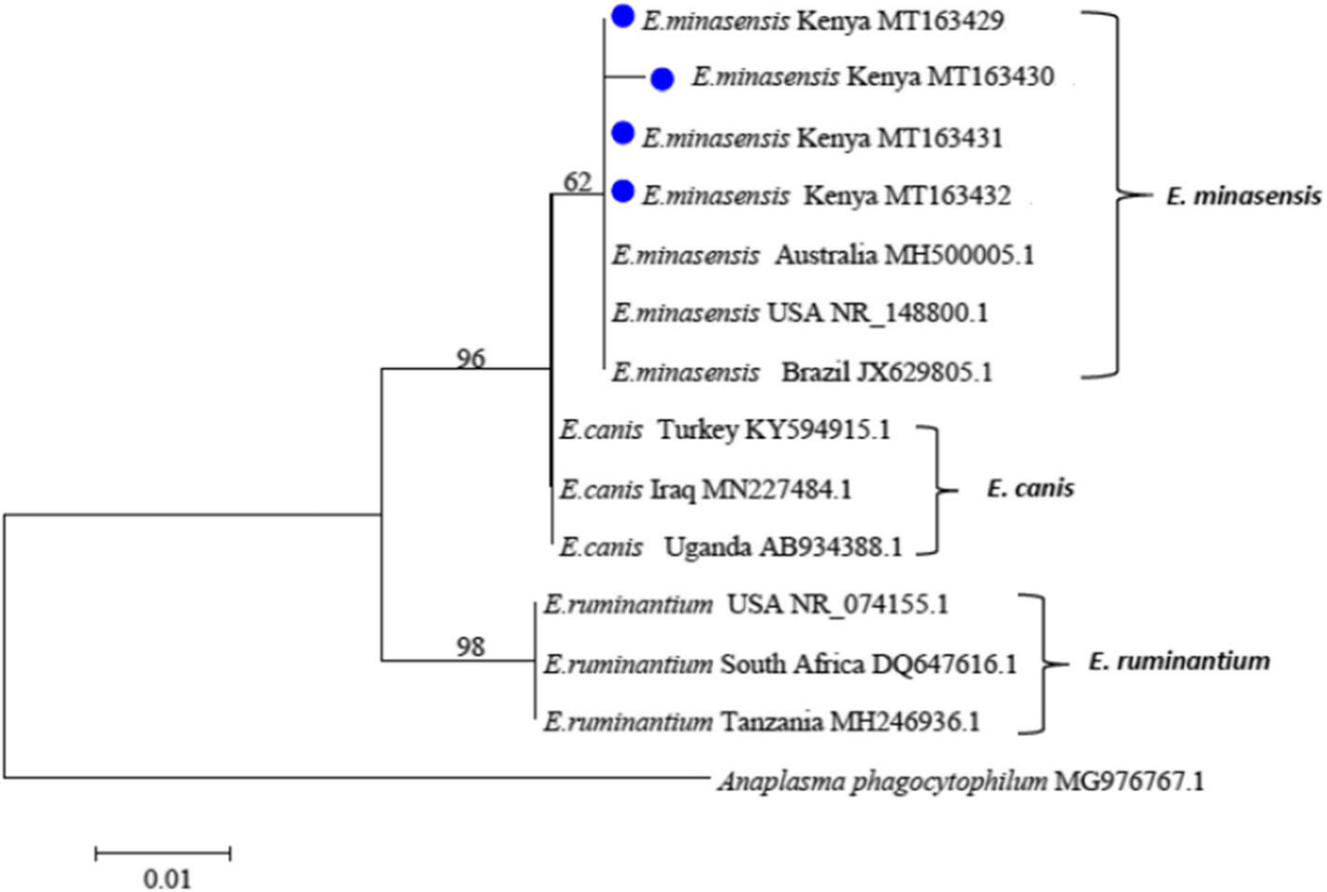
Maximum Likelihood tree of *Ehrlichia* spp. constructed using partial sequences of 16S rDNA gene. The tree is drawn to scale, with branch lengths measured in the number of substitutions per site. The analysis involved 4 nucleotide sequences from this study and 10 others obtained from Genbank. The phylogeny shows the relatedness of *E. minasensis* isolated from this study marked with blue dot with other isolates from USA, Brazil and Australia and its relation to *E. canis* and *E. ruminantium*. *Anaplasma phagocytophilum* was used as the outgroup

Phylogenetic analysis was done to understand genetic relatedness of the Kenyan isolates of the two genera with those of annotated sequences in GenBank (Figs. 3 and 4). The Kenyan isolates of *A. platys* clustered in the same clade as those of *A. platys* isolated from South Africa, Nigeria and Iran. They were however distinct from an isolate from India accession number MG711856.1 (Fig. 3-Clade 1). The Kenyan isolates of *A. marginale* were closely related to those from Uganda, USA, Australia and Iran (Clade 3). *Anaplasma bovis* isolates from Kenya were closely related to those from China but distantly related to those from South Korea and Japan (Clade 2). The unidentified *Anaplasma* species from this study clustered in their own clade separate from *A. platys*, *A. marginale* and *A. bovis* (Clade 4). For the *Ehrlichia* species, phylogeny was done to compare the detected *E. minasensis* genetic relatedness to other characterized species such as *E. canis*, the dog pathogen and the more common ruminant pathogen, *E. ruminantium.* The *E. minasensis* isolated in this study grouped in one clade with other isolates from USA, Australia and Brazil. These isolates were however closely related to *E. canis* than *E. ruminantium* (Fig. 4).

## Discussion

This study aimed at detecting and characterizing *Anaplasma* and *Ehrlichia* species infecting dairy cattle in peri-urban Nairobi. This information is important in guiding development of control and preventive measures against the infections caused by these tick-borne pathogens.

Overall, we observed more *Anaplasma* infections than *Ehrlichia* in the study cattle population. Previous studies in Kenya [7], Sudan [24] and Ethiopia [25] also reported more *Anaplasma* pathogens than *Ehlichia.* This could be explained by the wide diversity of *Anaplasma* compared to *Ehrlichia* species that can potentially infect cattle [3]. Moreover, spatial occurrence of tick-borne pathogens has been associated with the presence of their tick vectors [26], so it could be that tick vectors which transmit *Anaplasma* species are more widespread in the study area than those of *Ehrlichia* pathogens.

The distribution of the infections varied across sample sub-counties. A higher proportion of cattle were infected with *Anaplasma* and *Ehrlichia* spp. in Kasarani-Ruai Sub-County compared to the other three study areas of Dagoretti, Westlands and Lang’ata. A possible explanation of this is that dairy farmers in Kasarani-Ruai practice mixed production system involving free and zero grazing unlike the other areas where farmers practiced exclusive zero-grazing. Various studies have shown that free grazing cattle have higher risk of tick-borne infections than zero grazed cattle because of high exposure to tick vectors [22].

*Anaplasma platys* pathogens were detected in the study cattle. *Anaplasma platys* has been considered an emerging *Anaplasma* species whose clinical disease is yet to be described [9, 27]. Previous studies in Algeria [2], Senegal [27] and Tunisia [28] similarly reported this pathogen in cattle. Yang et al. [29] suggested a possibility of domestic ruminants acting as alternative hosts or reservoirs for *A. platys* which is typically a canine pathogen [30]. Therefore, the detection of this pathogen in cattle raises questions of host specificity as earlier speculated [31].

Zobba et al. [9] noted that several domestic ruminants can harbor a number of strains of *A. platys* although these strains have different cell tropism compared to those infecting dogs. The ruminant strains infect neutrophils and are thought to be the ancestral pathogens that evolved to adopt to the canine platelets instead [9, 30]. We think that infection with *A. platys* may be associated with co-existence of dogs and cattle in the same households, a common practice observed in the dairy farms in peri-urban Nairobi. It is possible that tick-bites from *Rhipicephalus sanguineus* which are the main vectors of *A. platys* [32] may have played a role in the transmission. Screening of dogs for this pathogen can reveal if they are acting as maintenance hosts of the parasite.

Previous studies have documented the zoonotic potential of *A. platys* causing human disease characterized by headaches, intermittent edema and muscle pains [33]. In this regard, detection of *A. platys* pathogens in this study would indicate a possible zoonotic health risk to cattle owners who are in constant contact with their cattle.

In this study, cattle were also found to be infected with *A. marginale*, a common pathogen of cattle that has been reported in Eastern Africa [22], Southern Africa [34], North Africa [27] and West Africa [32]. It is not surprising to detect this pathogen in cattle in Kenya since *Rhipicephalus (boophilus)* tick species which are the documented vectors of *A. marginale* are widespread in Kenya. *Anaplasma marginale* causes a mild to severe anemia depending on the susceptibility of the cattle [35]. However, cattle in this study were apparently healthy suggesting a possible endemic situation or the animals had a persistent infection (PI) state which is known to occur in *A. marginale* infections [36]. Thus, infected animals can appear apparently healthy despite harboring the pathogen.

*Anaplasma bovis* which is a monocytic pathogen of ruminants was also detected in this study. Different tick species in the genera *Amblyomma* and *Rhipicephalus* have been documented to transmit this pathogen [6]. Similar studies have detected this pathogen in Kenya [7], China [37], South Korea [3] Tunisia [38] and Algeria [39]. Despite it causing a mild disease in cattle, some infected animals have been shown to manifest with decreased weight gain, fever and lymphadenopathy [40]. Other studies have documented sub-clinical infection with this parasite where animals don’t show clinical signs of the disease despite the infection [41] and this may have been the case in our study.

To date, bovine anaplasmosis in Kenya is mainly known to be caused by *A. marginale* and to some extent *A. bovis* [7, 16, 22]. However, we have detected *A. platys* pathogens for the first time in cattle in Kenya possibly contributing equally to the disease burden in dairy cattle. Further studies to investigate this pathogen using more specific genes such as membrane surface proteins (Msps) [42] are justified.

An emerging pathogen *E. minasensis* in the *Ehrlichia* genera was also detected in this study. This novel pathogen was initially reported in Canada [43] and Brazil [44] but has since been isolated in Ethiopia [45], South Africa [46], Pakistan [47] and China [48]. The clinical disease due to *E. minasensis* is variable with some reports of severe disease [14] and at times sub-clinical disease as observed in this study [45]. Screening of ticks collected from animals may confirm if the pathogen is common in cattle.

Although the specific ticks that transmit *E. minasensis* have not been well studied [49], its detection and transstadial transmission by *Rhipicephalus microplus* ticks has been documented [50]. Other tick species may still transmit the pathogen in areas where *R. microplus* is absent [45]. Indeed, Iweriebor et al. [46] detected this pathogen from *R. appendiculatus, R. evertsi eversi, R. sanguineus* and *Ambylomma hebraeum* ticks. Some of these tick species were observed to be infesting some animals in this study. It is possible that these ticks could be involved in transmission of this pathogen.

Similar to other studies [38, 51], phylogenetic analysis based on 16S rDNA was used to infer genetic diversity of *Anaplasma* and *Ehrlichia* species. However, other authors have used 16S rDNA gene in combination with other genes such as heat shock protein (groEL), citrate synthase (gltA), 23S rDNA and major surface protein 4 gene (msp4) [2, 52]. Combined gene assays have been used to enhance sensitivity since genes with multiple copies such as membrane surface proteins (MSP) are more sensitive for detection of Anaplasmataceae while more conserved genes (16S rDNA) are useful for database cross matching and sequence comparisons [53]. In this study, *A. marginale* isolates were found to be highly conserved indicating sequence divergence of less than 2.5% and clustered together with those from USA, Uganda, Iran and Australia similar to findings by Rjeibi et al. [39]. *Anaplasma bovis*, *A. platys* pathogens and *E. minasensis* strains detected in this study indicated certain levels of nucleotide polymorphism suggesting various strains of the pathogens may exist in the study cattle. This may be related to the increased cattle movement from other regions of the country for slaughter at the country’s major export abbatoirs located in Nairobi County. Extensive animal movement has been associated with development of new strains and introduction of the tick-borne pathogens to new geographic areas [38, 54].

In agreement with previous studies, phylogenetic analysis also indicated that *E. minasensis* is closely related to *E. canis* and distantly related to *E. ruminantium* despite infecting similar hosts [12, 55]. The clinical presentation of *E. minasensis* in cattle has been observed to be similar to the acute form of disease by *E. canis* in dogs [44, 56]. Cabezas-Cruz et al. [57] links the close relatedness of the two pathogens to possible evolution of *E. minasensis* from highly variable strains of *E. canis*. The detection of this novel *Ehrlichia* species suggests that it could be circulating in cattle in Kenya and its pathogenicity in the affected animals needs to be determined.

Despite the animals in this study not presenting with the clinical signs of the diseases caused by the pathogens they harbor, poor animal husbandry practices which are commonly practiced in smallholder dairy farms in peri-urban areas of Nairobi [58], causes stress to the animals consequently predisposing them to possible flaring up of clinical disease and mortalities [59]. The detection of these pathogen therefore highlights the importance of continued investigation into tick-borne diseases and the need for effective diagnosis and prevention.

## Conclusion

The dairy cattle from peri-urban Nairobi, Kenya are infected with a range of *Anaplasma* species and *Ehrlichia minasensis* even though clinical disease was not evident. There is need for accurate diagnosis and effective tick control so as to reduce infection of cattle with these pathogens. To the best of our knowledge, this study provided first reports of cattle infected with *A. platys* and *E. minasensis* pathogens in Kenya. Extensive epidemiological studies would be necessary to determine the extent and pathogenicity of the newly detected *A. platys* and *E. minasensis* in cattle. Moreover, investigation into tick vectors involved in their transmission will be needed to inform strategic disease management and control. Moreover, there is need for further investigation of the unidentified *Anaplasma* species using more specific genes such as membrane surface proteins (Msps) [42].

## Methods

### Study area and design

The study area and design have been described in detail previously [23]. This was a cross-sectional study undertaken between January and May 2017. For purposes of data collection, Nairobi County was divided into four quadrants and for each quadrant purposive sampling was used to select the sub-county with high cattle population. Dairy farms in the four sub-counties in peri-urban Nairobi, namely; Dagoretti, Lang’ata, Kasarani-Ruai and Westlands were included to this study. These sub-counties are part of the peri-urban areas of the Nairobi County where dairy production has been established to meet the high milk demands of the urban population. Animals of different age-groups ranging from 3 months to 8 years were randomly selected and sampled for whole blood.

### Collection of cattle blood samples

Three milliliter of whole blood were collected in ethylenediaminetetraacetic acid (EDTA)-coated vacutainers from the coccygeal vein of 306 apparently healthy dairy cattle. The samples were collected as follows: Dagoretti (*n* = 116), Lang’ata (*n* = 55), Kasarani-Ruai (*n* = 110) and Westlands (*n* = 25). The samples were then transported in an ice-box to the Molecular Laboratory at the Department of Public Health, Pharmacology and Toxicology of the University of Nairobi and stored at -20 °C pending subsequent analysis.

### Extraction of *Anaplasma* and *Ehrlichia* DNAs

Whole blood genomic DNA (gDNA) was extracted from aliquots of 200 μl using QIAamp DNA Blood Mini Kit (Qiagen, Hilden, Germany) following manufacturer’s instructions. The DNA concentration and quality were assessed using QIAxpert (Qiagen, Hilden, Germany). DNA samples were then stored at − 20^0^ C.

### Primers design and PCR-amplification of *Anaplasma* and *Ehrlichia* DNA

A forward primer, ANAF 5′-TAGTGGCAGACGGGTGAGTA-3′ and a reverse ANAR 5′-AATTCCGAACAACGCTTGCC-3′ targeting an approximately 424 bp of *Anaplasma* 16S rDNA were designed using the Primer-BLAST tool of the National Center for Biotechnology Information [60] (NCBI) (www.ncbi.nlm.nih.gov/tools/primer-blast).

A forward primer EHRF 5′-AGCTGGTCTGAGAGGACGAT-3′ and a reverse primer EHRR 5′-GAGTGCCCAGCATTACCTGT-3′ targeting an approximately 838 bp of *Ehrlichia* 16S rDNA were also designed. PCR amplifications were performed using a thermal cycler (Applied biosystems Veriti 96 well, ThermoFisher). The *Anaplasma* and *Ehrlichia* 16S rDNA were amplified in a final volume of 20 μl reaction, each containing 3 μl of genomic DNA, 10 μl Master-mix (Taq PCR 2x mastermix, Qiagen, Germany) and 10 μM final concentration of each primer. The thermocycling conditions for *Anaplasma* involved a pre-denaturation at 95^o^ C for 5 min followed by 40 cycles of denaturation at 95^o^ C for 45 s, annealing at 57^o^ C for 45 s and extension at 72^o^ C for 45 s. A final cycle of extension at 72 °C for 7 min was performed. The amplification conditions for *Ehrlichia* 16S rDNA involved an initial denaturation cycle at 95^o^ C for 5 min followed by 35 cycles of denaturation at 95 °C for 45 s, annealing at 62^o^ C for 45 s and extension at 72^o^ C for 45 s. The amplification cycles were followed by a final cycle of extension at 72^o^ C for 7 min. Double distilled water was used as negative control for both assays. The amplified products were electrophoresed using 1.5% agarose gel in Tris-Borate-EDTA (TBE) buffer, pH 8, stained with Ethidium Bromide and visualized using UV-illuminator (GelMax® Imager, UK). The sizes of the amplicons were determined using molecular ladder (Gelpilot 1 kb plus ladder (100), Qiagen, Germany).

### Purification and sequencing of *Anaplasma* and *Ehrlichia* DNA

PCR amplicons in gels were excised and purified using QIAquick Gel Extraction Kit (Qiagen, GmbH, Germany) following the manufacturer’s protocol. The purified DNA were sequenced at Macrogen Europe Laboratories (Amsterdam, The Netherlands). Sequencing was done using the same forward and reverse primers as for the PCR reactions. The obtained sequences were viewed and manually verified using chromatogram peaks, edited and assembled using CLC Main Workbench 6.8.3 software (CLC bio, Qiagen GmbH, Germany).

### Data analysis

Bioinformatics analysis of the parasite 16S rDNA sequences was done by using Basic Local Alignment Search Tooln (BLASTn), multiple sequence alignment and phylogenetic analyses. Sequence identities of the *Anaplasma* and *Ehrlichia* species were confirmed by BLASTn analysis [61] at https://blast.ncbi.nlm.nih.gov/Blast.cgi. Multiple sequence alignment was done using Log-Expectation (MUSCLE) v3.8.31 [62]. Sequence similarity was calculated using Clustal Omega to obtain identity matrixs [63]. A phylogenetic reconstruction was done using MEGA 6.0 [64]. The evolutionary history was inferred by using the Maximum Likelihood method based on the Tamura-Nei model [65]. Initial trees for the heuristic search were obtained automatically by applying Neighbor-Join and BioNJ algorithms to a matrix of pairwise distances estimated using the Maximum Composite Likelihood (MCL) approach and then selecting the topology with superior log likelihood value. All positions containing gaps and missing data were eliminated. The percentage of replicate trees in which the associated taxa clustered together in the bootstrap test (1000 replicates) was shown next to the branches.

### Nucleotide sequence accession numbers

The partial 16S rDNA sequences obtained in this study were deposited in the GenBank under the following accession numbers; MT163376 to MT163388 for *A. platys*, MT160355 to MT160358 for *A. bovis*, MT163438 to MT163446 for *A. marginale*, MT163683 to MT163685 for unidentified *Anaplasma* species and MT163429 to MT163432 for *E. minasensis*.

## Data Availability

Dataset used and analyzed during the current study are available from the corresponding author on reasonable request. Sequences obtained and analyzed from this study are available in GenBank database on the accession numbers indicated.

## Abbreviations

TBDs: Tick-borne diseases
CI: Confidence Interval
ELISA: Enzyme-linked immunosorbent Assay
BLAST: Basic Local Alignment Search Tool
MEGA: Molecular Evolutionary Genetics Analysis
PI: Persistent Infection
